# Pertuzumab in combination with trastuzumab and chemotherapy for Chinese patients with HER2-positive metastatic gastric or gastroesophageal junction cancer: a subpopulation analysis of the JACOB trial

**DOI:** 10.1186/s40880-019-0384-6

**Published:** 2019-06-24

**Authors:** Tianshu Liu, Yanru Qin, Jin Li, Ruihua Xu, Jianming Xu, Shujun Yang, Shukui Qin, Yuxian Bai, Changping Wu, Yixiang Mao, Haiyan Wu, Yilin Ge, Lin Shen

**Affiliations:** 1 Department of Oncology, Zhongshan Hospital, Fudan University, Shanghai, 200032 P. R. China; 2 Department of Clinical Oncology, The First Affiliated Hospital, Zhengzhou University, Zhengzhou, 450052 Henan P. R. China; 3 Department of Medical Oncology, Fudan University Shanghai Cancer Center, Shanghai, 200032 P. R. China; 4 Department of Oncology, Tongji University Eastern Hospital, Shanghai, 200120 P. R. China; 5 Department of Medical Oncology, Sun Yat-Sen University Cancer Center, Guangzhou, 510060 Guangdong P. R. China; 6 Department of Oncology, 307th Hospital of PLA, Academy of Military Medical Sciences, Beijing, 100071 P. R. China; 7 Department of Medical Oncology, Affiliated Cancer Hospital of Zhengzhou University, Henan Cancer Hospital, Zhengzhou, 450008 Henan P. R. China; 8 Department of Oncology, People’s Liberation Army (PLA) Cancer Center, 81st Hospital of PLA, Nanjing, 210002 Jiangsu P. R. China; 9 Medical Department, Harbin Medical University Cancer Hospital, Harbin, 150081 Heilongjiang P. R. China; 10 Department of Oncology, First People’s Hospital of Changzhou, Changzhou, 213003 Jiangsu P. R. China; 11 Roche (China) Holding Co., Ltd., Shanghai, 201203 P. R. China; 12 Medical Division, Shanghai Roche Pharmaceuticals Ltd., Shanghai, 201203 P. R. China; 13 Key Laboratory of Carcinogenesis and Translational Research (Ministry of Education/Beijing), Department of Gastrointestinal Oncology, Peking University Cancer Hospital and Institute, #52 Fucheng Road, Haidian District, Beijing, 100142 P. R. China

**Keywords:** Gastric cancer, Gastroesophageal junction cancer, HER2, Pertuzumab, Trastuzumab, Chemotherapy, Overall survival, Progression-free survival, Safety, China

## Abstract

**Background:**

The JACOB trial (NCT01774786) was a double-blinded, placebo-controlled, randomized, multicenter, international, phase III trial evaluating the efficacy and safety of adding pertuzumab to trastuzumab and chemotherapy in first-line treatment of human epidermal growth factor receptor 2 (HER2)-positive metastatic gastric cancer/gastroesophageal junction cancer (GEJC). The aim of this analysis was to investigate efficacy and safety outcomes in the Chinese subpopulation from the JACOB trial.

**Methods:**

This post hoc subpopulation analysis included all patients recruited in mainland China (*n *= 163; 20.9%) between June 2013 and January 2016. The patients were randomly assigned in a 1:1 ratio to receive pertuzumab plus trastuzumab and chemotherapy (pertuzumab group; *n *= 82) or placebo plus trastuzumab and chemotherapy (control group; *n *= 81). Intravenous pertuzumab (840 mg) and trastuzumab (8 mg/kg loading and 6 mg/kg maintenance doses) were given every 3 weeks until disease progression or unacceptable toxicity. Chemotherapy was given as per standard regimens/doses of capecitabine or 5-fluorouracil plus cisplatin. The primary endpoint was overall survival (OS); secondary efficacy endpoints included progression-free survival (PFS), and overall objective response rate (ORR).

**Results:**

The median OS was 18.7 months in the pertuzumab group and 16.1 months in the control group (hazard ratio [HR] 0.75; 95% confidence interval [CI] 0.49 to 1.14). The median PFS was 10.5 and 8.6 months in the pertuzumab and control groups, respectively (HR 0.85; 95% CI 0.60 to 1.21), and the median ORRs were 68.9% and 55.7%, respectively. The treatment effect in this Chinese subpopulation showed consistency with that in the global ITT population with numerically lower HR for OS and PFS compared with the control group. The safety profiles of the pertuzumab and control groups in this Chinese subpopulation analysis were generally comparable. The most common grade 3–5 adverse events were neutropenia, anemia, and leukopenia. However, due to the nature of being a post hoc subgroup analysis, the results presented here are descriptive only and need to be interpreted with caution.

**Conclusions:**

OS and PFS were numerically improved by adding pertuzumab to trastuzumab and chemotherapy as first-line treatment in Chinese HER2-positive gastric cancer/GEJC patients, and this regimen demonstrated an acceptable safety profile.

*Trial registration* ClinicalTrials.gov. NCT01774786. Registered on 24 January 2013, https://clinicaltrials.gov/ct2/show/NCT01774786

**Electronic supplementary material:**

The online version of this article (10.1186/s40880-019-0384-6) contains supplementary material, which is available to authorized users.

## Background

Gastric cancer is the fifth most common cancer and the third leading cause of death worldwide [1]. More than 70% of new gastric cancer cases occur in less developed countries; furthermore, 42.6% of new cases and 45.0% of gastric cancer-related deaths occur in China [2]. In China, gastric cancer is the second most common cancer as well as the second leading cause of cancer-related death, after lung cancer [2]. Approximately 679,100 new gastric cancer cases were diagnosed and 498,000 cancer-related deaths were reported in 2015, accounting for 15.8% of total cancer incidence and 17.8% of cancer mortality in China [3]. Thus, the disease burden owing to gastric cancer is much higher in China than elsewhere, and urgent efforts are required to improve the preventative measures and treatments for gastric cancer [1].

The main reason for the poor prognosis of gastric cancer is that the majority of patients present with advanced or inoperable disease at the time of diagnosis, resulting in limited available treatment options. The lack of a formal, nationwide, gastric cancer screening program has contributed to a lower early detection rate in China compared with those in other Asian countries; the 5-year overall survival (OS) rate for Chinese patients with gastric cancer has been reported to be 54.3%, and only 15.0% for patients with distal metastasis [4]. Short survival rates were also reported previously in the analysis of data from the China subpopulation of the phase III ToGA study [5]. Although long-term survival rates have been improved in recent years [6], possibly due to heightened health awareness as well as the implementation of new treatment strategies and follow-up procedures, they still remain lower in China than in other Asian countries [4, 7, 8]. It is clear that more needs to be done to improve the outcomes for these patients.

Many investigations on new treatment strategies focus on advanced disease, including those targeting human epidermal growth factor receptor 2 (HER2), a molecular target with demonstrated treatment benefits in HER2-positive gastric cancer [9]. In the ToGA study, trastuzumab combined with a fluoropyrimidine [capecitabine or 5-fluorouracil (5-FU)] plus cisplatin was found to be more effective and tolerable in HER2-positive advanced gastric cancer and gastroesophageal junction cancer (GEJC) compared with placebo plus chemotherapy [5, 10]. In a post hoc analysis conducted on patients whose tumors highly overexpressed HER2, a 35% reduction in the risk of death (hazard ratio [HR] 0.65; 95% confidence interval [CI] 0.51–0.83) was observed with a median overall survival (OS) of 16.0 months in the treatment arm, compared with 11.8 months in the control arm [11]. A subpopulation analysis of Chinese patients produced results consistent with those obtained for the global population; as a result, trastuzumab plus chemotherapy was approved for use in HER2 immunohistochemistry (IHC)3+ or IHC2+ and in situ hybridization (ISH)-positive metastatic gastric cancer in China in 2012 [12]. To date, other phase III trials of first-line treatments have not achieved positive results [13–17]. Thus, trastuzumab plus chemotherapy is currently the only available targeting regimen for first-line treatment of HER2-positive metastatic gastric cancer. Nonetheless, improvements in median OS are still required.

The emergence of pertuzumab, a HER2 dimerization inhibitor and a new class of targeted agent, provides a new treatment option. Pertuzumab and trastuzumab bind to distinct sites on HER2, without competing with each other, and therefore disrupt HER2 signaling via complementary mechanisms [18]. Based on a series of promising data [19–21], the JACOB study was designed as a phase III trial with the hypothesis that pertuzumab combined with trastuzumab and chemotherapy as a first-line treatment can augment anti-HER2 activity to prolong the OS of patients with HER2-positive metastatic gastric cancer/GEJC [22].

The primary analysis of the JACOB study was performed based on the clinical cut-off date of December 9, 2016 [22]. JACOB did not meet its primary endpoint; however, the addition of pertuzumab was found to numerically prolong the median OS by 3.3 months (17.5 months in the pertuzumab group vs. 14.2 months in the control group), although this difference was not statistically significant (HR 0.84; 95% CI 0.71 to 1.00; *P* = 0.0565) [22]. Moreover, 20.9% (*n* = 163) of all participants enrolled in the study were recruited from mainland China. The purpose of conducting the current post hoc subpopulation analysis was to investigate the efficacy and safety in Chinese patients with HER2-positive, metastatic gastric cancer and GEJC.

## Methods

### Study design and treatment

The JACOB trial was a double-blinded, placebo-controlled, randomized, multicenter, international phase III trial designed to evaluate the efficacy and safety of pertuzumab in combination with trastuzumab and chemotherapy as a first-line treatment in patients with HER2-positive metastatic gastric cancer or GEJC. The primary results of an intention-to-treat (ITT) population, which was defined as all patients randomly assigned to treatment groups regardless of whether they received the study drug, were analyzed according to group allocation have already been published [22]. Briefly, the HER2-positive patients were histologically confirmed with metastatic adenocarcinoma of the stomach or gastroesophageal junction. HER2-positive tumors were defined as primary or metastatic tumors with either IHC3+ or IHC2+ plus ISH+, as assessed by a sponsor-designated central laboratory (Targos Molecular Pathology GmbH, Kassel, Germany or Q-Lab, Shanghai, China). Other criteria were an Eastern Cooperative Oncology Group performance status (ECOG PS) of 0 or 1, baseline left ventricular ejection fraction (LVEF) of ≥ 55%, and a life expectancy of ≥ 3 months. In total, 780 patients (388 patients in the pertuzumab group and 392 patients in the control group) were enrolled at 197 centers across 30 countries between June 10, 2013 and January 12, 2016 [22].

Eligible patients were randomized in a 1:1 ratio to the pertuzumab group or the control group. Patients were stratified based on geographical region (Asia [excluding Japan], Japan, North America/Western Europe/Australia, and South America/Eastern Europe), prior gastrectomy (yes and no), and HER2 status (IHC3+ and IHC2+/ISH+); the geographical region of China was not one of the stratification factors for the main study. The patient number and treatment assignment were provided to the investigator via the interactive voice or Web response system (IxRS; Almac Group, Souderton, PA, USA). Patient randomization numbers were allocated sequentially in the order in which patients were enrolled. The pertuzumab group received pertuzumab (840 mg by intravenous [IV] injection every 3 weeks) plus trastuzumab (IV, 8 mg/kg loading dose on day 1, followed by 6 mg/kg every 3 weeks) plus chemotherapy (cisplatin 80 mg/m^2^ by IV every 3 weeks; capecitabine 1000 mg/m^2^ taken orally twice daily for 28 doses every 3 weeks or 5-FU 800 mg/m^2^ daily by continuous IV infusion for 120 h every 3 weeks). The control group received placebo plus trastuzumab plus chemotherapy (regimens as above). The type of chemotherapy administered was determined by the treating physician. Chemotherapy was discontinued only for progressive disease or unacceptable toxicity (assessed using the National Cancer Institute Common Terminology Criteria for Adverse Events [NCI-CTCAE] version 4.0) during or before the 6th cycle. After the 6th cycle, continuation of chemotherapy was at the discretion of the patients and the treating physician. After chemotherapy completion, all patients received pertuzumab and trastuzumab or placebo and trastuzumab until disease progression, unacceptable toxicity, or withdrawal from the study. The study was considered to have reached completion when OS data for 502 events were collected and the last patient who received the study treatment had completed 5 years of cardiac safety follow-up or when the study was terminated by the sponsor, whichever occurred first.

This study was conducted in full conformance with the International Conference on Harmonization’s Guideline for Good Clinical Practice (document E6) [23] and the principles of the Declaration of Helsinki. All patients provided signed written informed consent. The study was registered at ClinicalTrial.gov (NCT01774786).

### Endpoints and measurements

The primary objective was to compare OS, defined as the time from randomization to death of any cause or until the date of last follow-up, between the pertuzumab group and the control group. The secondary efficacy endpoints were progression-free survival (PFS), overall objective response rate (ORR), and duration of response (DoR). PFS was defined as the time from randomization to the first occurrence of disease progression, as determined by the investigator using RECIST v1.1 [24], or until death of any cause. ORR was defined as the percentage of patients who achieved either a partial response (PR) or complete response (CR) as determined using RECIST v1.1 based on the investigators’ assessment and was confirmed (using computed tomography, magnetic resonance imaging, or X-ray) on two consecutive occasions ≥ 4 weeks apart after the criteria for response were first met. DoR was defined as the time from the date of the first documented objective response to the date of first documented progressive disease (PD) or death, whichever occurred first. Safety outcomes included the rates of symptomatic left ventricular systolic dysfunction (LVSD), non-LVSD cardiac death, and probable cardiac death. The rate of asymptomatic LVSD, severity of adverse events (AEs), serious AEs, and other laboratory test abnormalities were measured over the course of the study. Symptomatic LVSD was defined as an absolute decrease from baseline of ≥ 10 % points in LVEF to a value of < 50% and at least one symptom of probable cardiac failure. Asymptomatic LVSD was defined as an absolute decrease in LVEF of ≥ 10 % points below the baseline measurement to an LVEF of < 50%.

### Post-hoc and statistical analyses

This post hoc subpopulation analysis included all patients recruited at centers in mainland China. The same analysis as that performed on the ITT population [22] was conducted, and descriptive statistics were provided without formal testing. For the assessment of OS, patients who remained in the study at the time of data cut-off were censored at the last date of/prior to cut-off when the patients were known to be still alive. Patients who did not have any post-baseline data were censored at the date of randomization plus 1 day. For the assessment of PFS and DoR, patients without documented PD or death at the end of the study were censored at the tumor assessment date for which the patient was last known to be progression-free. For the assessment of PFS, patients who did not have any post-baseline tumor assessment data were censored at the date of randomization plus 1 day. ORR was assessed for patients in the ITT population with measurable disease at baseline. Patients who did not have any post-baseline tumor assessment data were counted as non-responders. Safety outcomes were assessed in all randomized patients who received at least one dose of the study treatment (analyzed according to treatment received).

The median OS, PFS, and DoR for both groups were estimated using the Kaplan–Meier approach, with 95% confidence intervals (CIs) calculated using the Brookmeyer and Crowley method. An unstratified Cox proportional hazards regression model was used to estimate the hazard ratio (HR) between the two groups with 95% CI. The ORRs with 95% CIs were calculated for both groups using the Clopper–Pearson method. Statistical analyses were performed using the SAS software, version 9.2 and 9.4 (SAS Institute Inc., Cary, NC, USA).

## Results

### Patients

**Fig. 1:**
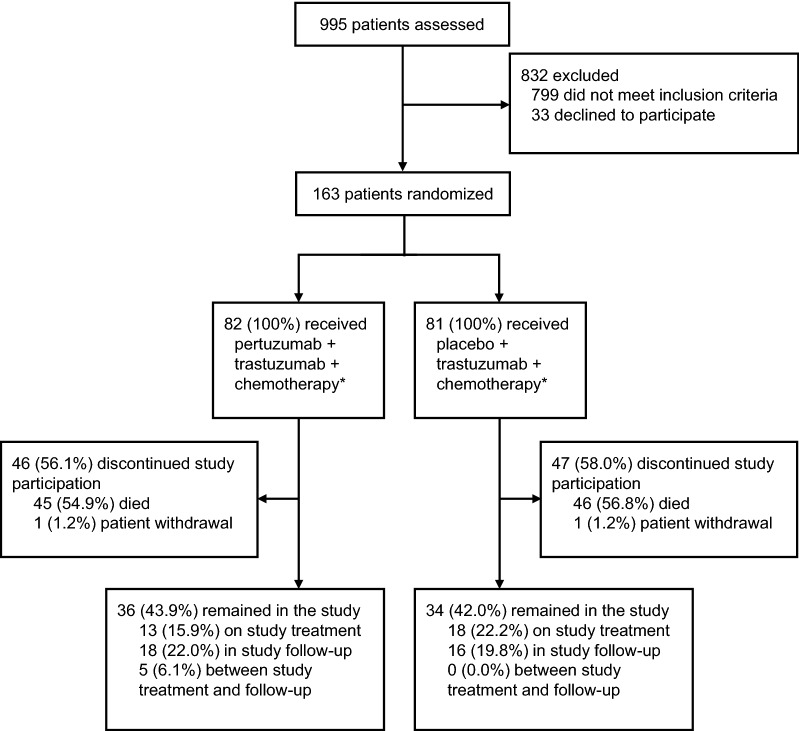
Study flowchart for the JACOB Chinese subpopulation. “Between study treatment and follow-up” refers to patients who have discontinued or completed study treatment but had not yet completed the first survival follow-up assessment. Patients in both treatment groups received trastuzumab, cisplatin, and fluoropyrimidine (capecitabine or 5-fluorouracil). *These patients were included in the intention-to-treat population. One patient assigned to the control group received one dose of pertuzumab in error and was included in the safety population for the pertuzumab group; the final safety population was consisted of 83 patients from the pertuzumab group and 80 patients from the control group

By the time of the primary clinical cut-off date of December 9, 2016, 995 Chinese patients were screened, and 163 patients were recruited at 23 centers across China between July 15, 2014 and December 30, 2015. The median duration of the study follow-up was 22 months (range 0–25 months) in the pertuzumab group and 18 months (range 2–26 months) in the control group. Following stratification according to prior gastrectomy and HER2 status, 82 patients were randomized to the pertuzumab group and 81 patients to the control group. All patients who were randomly assigned to treatment, regardless of whether the study medication was actually consumed, constituted the Chinese ITT subpopulation (Fig. 1). One patient who was randomized to the control group, but received pertuzumab, was included in the pertuzumab group for the safety analysis. Therefore, the safety analysis was based on 83 patients in the pertuzumab group and 80 patients in the control group. Only two patients, one in each group, withdrew after treatment (one patient received three cycles of treatment and discontinued treatment due to disease progression, then withdrew from the study; the other patient received one cycle of treatment before withdrawing from the study). No patients were lost to follow-up until the primary clinical cut-off date.

The patients’ baseline and clinical characteristics were comparable between the two groups (Table 1). The median age was 59 (range 25–78) years in the pertuzumab group and 59 (range 23–73) years in the control group. Females accounted for 28.0% of patients in the pertuzumab group and 12.3% in the control group. In total, 25.6% of patients in the pertuzumab group and 14.8% in the control group were diagnosed with GEJC. The average cycles of pertuzumab/placebo and trastuzumab administered per patient were 13.67 ± 9.36 in the pertuzumab group and 11.81 ± 8.35 in the control group. Relative dose intensity was similar in both groups, with a mean of 89.4% and 90.4% respectively.

**Table 1:** Baseline demographic and disease characteristics of the Chinese subpopulation of the JACOB trial (ITT population)

Characteristic	Total (cases)	Pertuzumab group [cases (%)]	Control group [cases (%)]
Total	163	82	81
Age			
< 65 years		61 (74.4)	62 (76.5)
≥ 65 years		21 (25.6)	19 (23.5)
Sex			
Male	130	59 (72.0)	71 (87.7)
Female	33	23 (28.0)	10 (12.3)
Disease measurability			
Measurable	144	74 (90.2)	70 (86.4)
Non-measurable	19	8 (9.8)	11 (13.6)
ECOG PS			
0	47	24 (29.3)	23 (28.4)
1	116	58 (70.7)	58 (71.6)
Histological subtype (Lauren classification)			
Diffuse	16	9 (11.0)	7 (8.6)
Intestinal	144	71 (86.6)	73 (90.1)
Mixed	3	2 (2.4)	1 (1.2)
Primary site			
Gastroesophageal junction	33	21 (25.6)	12 (14.8)
Stomach	130	61 (74.4)	69 (85.2)
Number of metastatic sites^a^			
1–2	139	68 (82.9)	71 (88.8)
> 2	23	14 (17.1)	9 (11.3)
HER status			
IHC2+ and ISH+	31	14 (17.1)	17 (21.0)
IHC3+	132	68 (82.9)	64 (79.0)
Prior gastrectomy			
Yes	47	23 (28.0)	24 (29.6)
No	116	59 (72.0)	57 (70.4)

*ITT* intention to treat; *ECOG PS* Eastern Cooperative Oncology Group performance status; *HER2* human epidermal growth factor receptor 2; *IHC* immunohistochemistry; *ISH* in situ hybridization

^a^One patient in the control group did not have data on the number of metastatic sites available

Regarding the chemotherapy regimen, capecitabine was administered to 72 (87.8%) patients in the pertuzumab group and 70 (86.4%) patients in the control group; 5-FU was administered to 14 (17.1%) patients in the pertuzumab group and 13 (16.0%) in the control group. Chemotherapy was the main post-progression therapy in the Chinese subpopulation, and the regimens used were similar between the two groups. In total, 25 (30.5%) patients in the pertuzumab group and 24 (29.6%) in the control group received at least one additional line of treatment subsequent to this study, and the details are shown in Additional file 1: Table S1.

### Efficacy

**Fig. 2:**
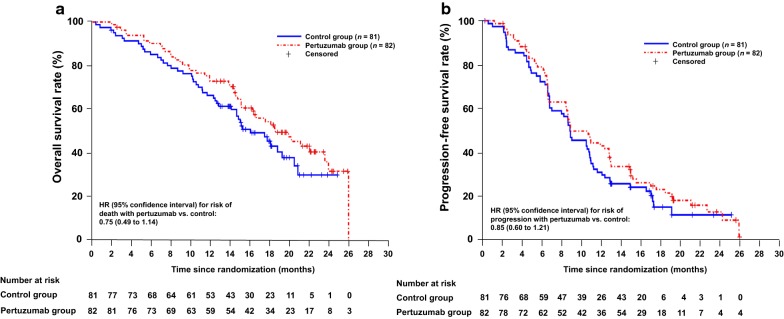
Kaplan–Meier plots of overall survival (**a**) and progression-free survival (**b**) in the Chinese intention-to-treat subpopulation. Hazard ratio (HR) values with 95% confidence intervals (CIs) are unstratified. Progression-free survival was assessed by the investigator. Patients in both treatment groups received trastuzumab, cisplatin, and a fluoropyrimidine (capecitabine or 5-fluorouracil)

In the Chinese subpopulation, the addition of pertuzumab to trastuzumab and chemotherapy reduced the risk of death by 25% compared with the control group (HR 0.75; 95% CI 0.49 to 1.14). The median OS was 18.7 months in the pertuzumab group, compared with 16.1 months in the control group (Fig. 2a). The median PFS was extended by approximately 2 months in the pertuzumab group (10.5 months vs. 8.6 months, HR 0.85; 95% CI 0.60 to 1.21) (Fig. 2b). The ORR was 68.9% (95% CI 57.1% to 79.2%) in the pertuzumab group compared with 55.7% (95% CI 43.3% to 67.5%) in the control group. The difference in ORR between the two groups was 13.2% (95% CI − 3.32 to 29.73).

### Safety

A total of 1278 and 1008 AEs were recorded in the pertuzumab group and the control group, respectively. The AE profile is shown in Table 2 and the most common AEs (≥ 10% patients in either treatment group) are shown in Table 3. The AE profiles of the two treatment groups were generally comparable except for diarrhea (all grades, 39.8% in the pertuzumab group vs. 16.3% in the control group). After causality assessment, the rates of treatment-related diarrhea were similar in both groups (all grades, 10.8% in the pertuzumab group vs. 10.0% in the control group). There was no treatment discontinuation due to diarrhea in the pertuzumab group. The three most common AEs (all grades) in both groups were neutropenia, leukopenia, and nausea, whereas the three most common grade 3–5 AEs in both groups were neutropenia, anemia, and leukopenia. Serious AEs occurred in 20.5% and 15.0% of patients in the pertuzumab and control groups, respectively. Three (3.6%, including 1 general physical health deterioration, 1 acute myocardial infarction, and 1 death) and 6 (7.5%, including 1 anemia, 1 septic shock, 1 respiratory failure, and 3 deaths) patients experienced AEs with fatal outcomes in the pertuzumab and control groups, respectively. All AEs with fatal outcomes in the pertuzumab group were assessed as unrelated to the study treatment. Treatment was discontinued because of AEs in seven (8.4%) patients in the pertuzumab group and five (6.3%) patients in the control group. No symptomatic LVSD was observed, and only one patient (1.2%) developed asymptomatic LVSD in the pertuzumab group.

**Table 2:** Summary of the adverse events that occurred in the two treatment groups (safety population)

Adverse event	Pertuzumab group [cases (%)] *n* = 83	Control group [cases (%)]*n* = 80
All-grade AEs	83 (100.0)	78 (97.5)
Grade 3–5 AEs	67 (80.7)	52 (65.0)
Serious AEs	17 (20.5)	12 (15.0)
AEs leading to treatment discontinuation	7 (8.4)	5 (6.3)
AEs with fatal outcome	3 (3.6)	6 (7.5)
AEs leading to study withdrawal	1 (1.2)	2 (2.5)

*AE* adverse event

**Table 3:** Listing of the most common adverse events occurring in ≥ 10% patients in either treatment group (safety population)

Most common AE	Pertuzumab group [cases (%)] *n* = 83		Control group [cases (%)] *n* = 80	
	Any grade	Grade 3–5	Any grade	Grade 3–5
Neutropenia	60 (72.3)	31 (37.3)	52 (65.0)	24 (30.0)
Leukopenia	53 (63.9)	14 (16.9)	47 (58.8)	12 (15.0)
Nausea	52 (62.7)	7 (8.4)	49 (61.3)	2 (2.5)
Anemia	50 (60.2)	20 (24.1)	43 (53.8)	18 (22.5)
Vomiting	38 (45.8)	9 (10.8)	36 (45.0)	6 (7.5)
Decreased appetite	37 (44.6)	3 (3.6)	29 (36.3)	1 (1.3)
Diarrhea	33 (39.8)	7 (8.4)	13 (16.3)	1 (1.3)
Thrombocytopenia	31 (37.3)	5 (6.0)	28 (35.0)	7 (8.8)
Fatigue	26 (31.3)	2 (2.4)	16 (20.0)	1 (1.3)
Hypokalemia	19 (22.9)	6 (7.2)	11 (13.8)	4 (5.0)
Abdominal distension	18 (21.7)	1 (1.2)	12 (15.0)	0
Palmar-plantar erythrodysesthesia syndrome	14 (16.9)	5 (6.0)	16 (20.0)	6 (7.5)
Creatinine renal clearance decreased	13 (15.7)	0	10 (12.5)	0
Constipation	11 (13.3)	0	12 (15.0)	0
Weight decreased	13 (15.7)	1 (1.2)	4 (5.0)	0
Stomatitis	14 (16.9)	3 (3.6)	7 (8.8)	1 (1.3)
Pyrexia	9 (10.8)	0	12 (15.0)	0

*AE* adverse event

## Discussion

In this post hoc subpopulation analysis, we present the efficacy and safety of pertuzumab in combination with trastuzumab plus chemotherapy in 163 Chinese patients with HER2-positive metastatic gastric cancer and GEJC recruited to the JACOB study. The treatment effect in this Chinese subpopulation showed consistency with that in the global ITT population with numerically lower HR for OS (HR 0.75; prolongation of median OS, 2.6 months) and PFS (HR 0.85; prolongation of median PFS, 1.9 months) compared with the control group. However, due to the nature of being a post hoc subgroup analysis, the results presented here are descriptive only and need to be interpreted with caution.

Differences in clinical and pathological characteristics were observed between the Chinese subpopulation and the global ITT population in certain aspects. There was a higher percentage of patients aged < 65 years in the Chinese subpopulation (74.4% in the pertuzumab group and 76.5% in the control group) compared with that in the global ITT population (58.8% in the pertuzumab group and 63.0% in the control group). A higher proportion of patients in the Chinese subpopulation had an ECOG PS of 1 (approximately 70% in each group) than that in the global ITT population (approximately 58%). Moreover, the diffuse histological subtype was observed in 9.8% of all patients in China, whereas 5.0% of patients in the global ITT population were diagnosed with this subtype.

Of note, HER2 IHC3+ patients accounted for 82.9% of the Chinese subpopulation, which was higher than that in the global ITT population (66.8%) [22]. It has been reported that the concurrence of HER2-positive status and diffuse subtype gastric cancer was associated with the worst survival outcomes [25]. All of these characteristics were in accordance with the known tumor biology and aggressiveness of HER2-positive metastatic gastric cancer in Chinese patients, with similar data observed in the ToGA [5], HELOISE [26], and EVIDENCE studies [27].

The long-term outcomes in the pertuzumab group were numerically better in the Chinese subpopulation than in the global ITT population from the JACOB trial. In addition, the HR for OS was numerically lower in the Chinese subpopulation than that in the global ITT population (0.75 vs. 0.84). The ORR also showed a similar trend of greater improvement in the Chinese subpopulation compared with the global ITT population (13.2% vs. 8.4%) [22]. These numerical differences between the Chinese subpopulation and global ITT population might be related to the unique baseline disease characteristics and post-progression therapy for Chinese patients. Chen et al. [28] reported that capecitabine plus cisplatin may result in PFS benefit compared with 5-FU plus cisplatin as first-line treatment for Chinese patients with advanced and metastatic GC. The discrepancies in efficacy endpoints between the Chinese subpopulation and the global ITT population might be associated with the fact that a greater proportion of Chinese patients receiving capecitabine (87.1% vs. 77.1% in the total global ITT population). However, we cannot rule out the possibility that it might also be resulting from confounding factors inherent from conducting a post hoc analysis of a subpopulation for which the original study was not specifically powered. It is hoped that future prospective studies will be able to more accurately ascertain this point.

The safety profile in the Chinese subpopulation was consistent with that in the global population of the JACOB trial and the known safety profile of pertuzumab. However, the nature of post hoc analyses and the lack of multiplicity control for subpopulation analyses require that all data presented here should be treated as descriptive statistics only. When comparing the safety profile of pertuzumab in this subpopulation with that in the global population, a lower frequency of diarrhea occurrence was observed. Of note, the same trend was observed in the Chinese subpopulation in the ToGA study [5].

The main limitations of our study were those inherent to post hoc subgroup analyses, including smaller sample size compared with the parent study, possibly reduced statistical power, less ability to control for confounding variables, and the potential bias in unblinded evaluations. Therefore, the results of this post hoc analysis should be interpreted with caution. Nonetheless, given the current absence of data from prospective clinical trials in Chinese patients, the results accruing from this analysis provide important preliminary information for clinicians to consider future research in patients with HER2-positive advanced gastric cancer.

## Conclusions

In summary, we here present the efficacy and safety results of first-line treatment with pertuzumab combined with trastuzumab plus chemotherapy in Chinese patients with HER2-positive, metastatic gastric cancer and GEJC. Compared with trastuzumab and chemotherapy alone, there was a numerically improved OS, PFS, and ORR, and a similar safety profile when pertuzumab was added to the treatment regimen. Clinically, these results demonstrated a good response to treatment in Chinese patients with HER2-positive, metastatic gastric cancer and GEJC, but further investigation is required to identify which patients are more likely to substantially benefit from pertuzumab treatment.

## Additional file

**Additional file 1: Table S1.** Post-study anti-cancer therapies used by at least 2 patients (ITT population).

## Data Availability

The datasets used and/or analyzed during the current study are available from the corresponding author on reasonable request.

## Abbreviations

HER2: human epidermal growth factor receptor 2
5-FU: capecitabine or 5-fluorouracil
GEJC: gastroesophageal junction cancer
HR: hazard ratio
OS: overall survival
IHC: immunohistochemistry
ISH: in situ hybridization
ITT: intention-to-treat
ECOG PS: Eastern Cooperative Oncology Group Performance Status
LVEF: left ventricular ejection fraction
PFS: progression-free survival
ORR: overall objective response rate
DoR: duration of objective response
PR: partial response
CR: complete response
PD: progressive disease
LVSD: left ventricular systolic dysfunction
AE: adverse event
CI: confidence interval
