# Supplementary material for: Pertuzumab in combination with trastuzumab and chemotherapy for Chinese patients with HER2-positive metastatic gastric or gastroesophageal junction cancer: a subpopulation analysis of the JACOB trial
Source: Cancer Commun (Lond). 2019 Jun 24;39:38. doi: 10.1186/s40880-019-0384-6 (PMC6591878; doi:10.1186/s40880-019-0384-6)
Supplement: Supplementary file 1 — Additional file 1: Table S1. Post-study anti-cancer therapies used by at least 2 patients (ITT population). [file 40880_2019_384_MOESM1_ESM.docx]

**Additional file 1: Table S1.** Post-study anti-cancer therapies used by at least 2 patients (ITT population)

| **Treatment type** | **Pertuzumab group [cases (%)]** | **Control group**  **[cases (%)]** |
| --- | --- | --- |
| All | 25 (30.5) | 24 (29.6) |
| Taxanes (docetaxel, paclitaxel) | 16 (19.5) | 18 (22.2) |
| Antimetabolites (tegafur, 5-fluorouracil, raltitrexed, capecitabine) | 10 (12.2) | 11 (13.6) |
| Platinum compounds (oxaliplatin, cisplatin) | 8 (8.8) | 4 (4.9) |
| Antineoplastic adjuncts (gimeracil, oteracil) | 5 (6.1) | 5 (6.2) |
| Topoisomerase inhibitors (irinotecan) | 3 (3.7) | 6 (7.4) |
| Tyrosine kinase inhibitors (apatinib, erlotinib, pyrotinib) | 2 (2.4) | 5 (6.2) |
| **Monoclonal antibodies (trastuzumab, pertuzumab, RC48)** | 3 (3.7) | 3 (3.7) |
| Herbal, homeopathic, or dietary supplements | 1 (1.2) | 3 (3.7) |
| Folic acid derivatives | 1 (1.2) | 2 (2.5) |

Abbreviations: *ITT*, intention to treat,
